# Insightful skiing: developing explainable models of on-snow performance through physical attribute selection of alpine skis

**DOI:** 10.1007/s12283-025-00511-w

**Published:** 2025-08-04

**Authors:** Jonathan Audet, ·Abdelghani Benghanem, ·Alexis Lussier‑Desbiens

**Affiliations:** 1 Createk Design Lab, Université de Sherbrooke, 3000 bd de l’Université, Sherbrooke, QC J1K 2R1 Canada; 2 Sooth Ski, 1234 William St, Quebec City, QC G1S 4E9 Canada

## Abstract

Evaluating alpine skis on snow is pivotal for ski development and consumer decision-making, yet it is resource-intensive and hindered by subjective assessments. Leveraging recent extensive ski physical measurements and on-snow ski evaluation metrics, this study proposes an automated methodology that employs elastic net regression, bootstrap resampling, and intelligent feature selection to predict the on-snow performance using a minimal set of physical attributes. Results on 192 skis divided into 10 categories and 29 metrics indicate promising predictive capabilities, with models exhibiting an average Mean Absolute Error rank prediction of 15%. Importantly, the models utilize less than three physical attributes on average, underscoring their simplicity and effectiveness in identifying key performance-defining properties. These findings, to the authors’ knowledge, represent the most comprehensive description of ski on-snow performance to date and hold implications for ski design and consumer guidance. Moreover, the automated methodology enables the easy integration of other evaluation sources, facilitating further refinement and validation, while promising to consider the diversity of opinions related to ski on-snow performance assessment.

**Supplementary Information:**

The online version contains supplementary material available at 10.1007/s12283-025-00511-w.

## Introduction

The on-snow evaluation of alpine skis is an important aspect of the ski industry [1, 2], serving to guide the development of the skis, as well as to inform skiers' and retailers’ purchase decisions through demo days and reviews. However, organizing and performing these evaluations requires a significant amount of effort, time, and expense. Moreover, the availability of suitable snow conditions during testing, as well as its variability throughout the evaluation period, and the human factors involved (e.g., physical characteristics and skills, bias, reliability within and across evaluators, fatigue) [3], pose challenges to ensure the reliable comparisons of the thousands of skis available each year. Finally, the results of these on-snow evaluations are often hard to interpret as they involve subjective performance criteria. These challenges typically limit the study of on-snow performance to 2–3 well-controlled design parameters, between 5 and 10 performance metrics, or a small number of skis (i.e., 4–12) [3–5]. The idea of developing general relationships that explain the on-snow performance of skis without the need for repeated field testing, therefore, holds great appeal to both the skier community and the ski industry.

**Fig. 1:**
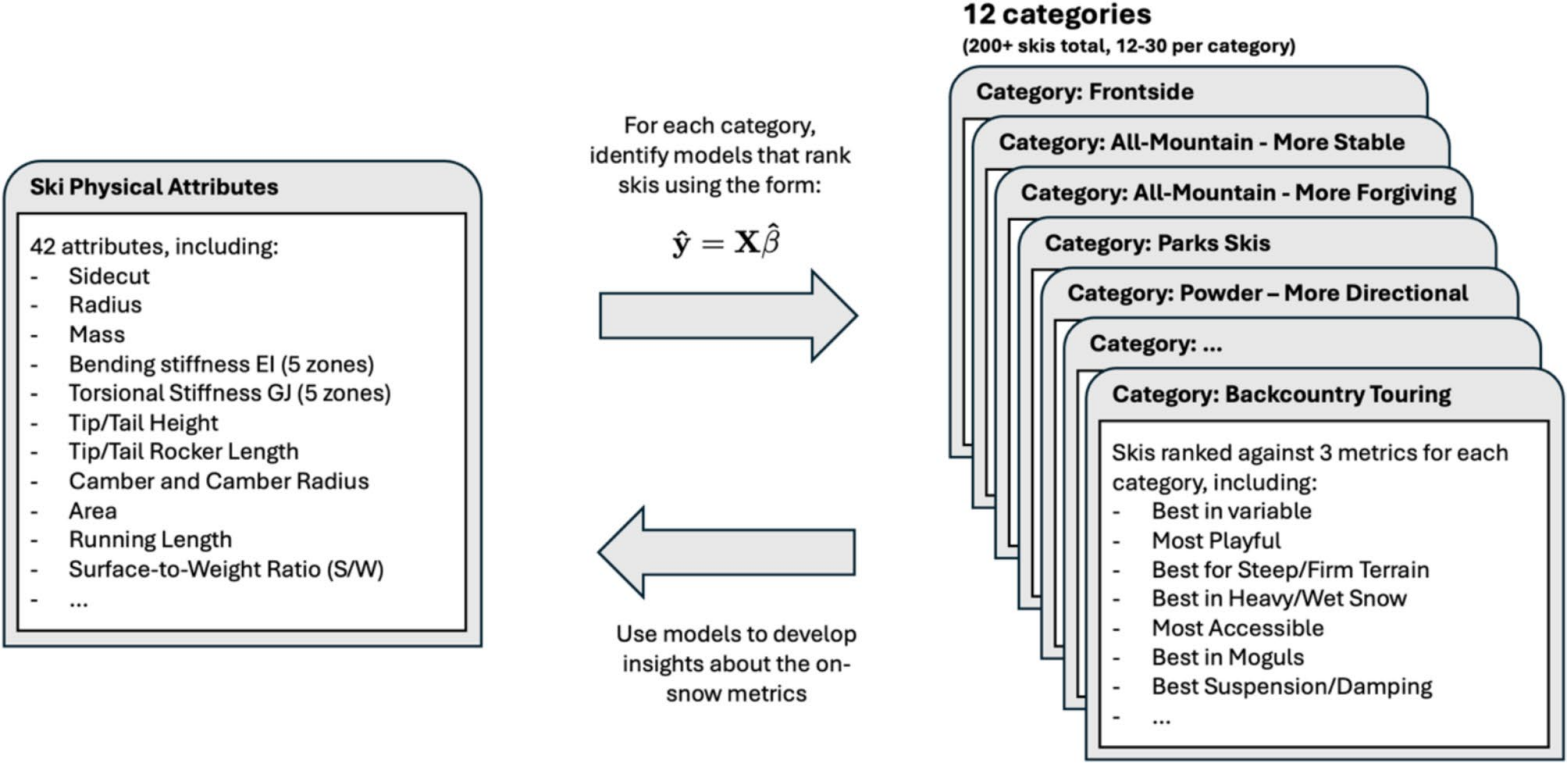
The proposed methodology aims at uncovering simple linear relationships between the skis’ on-snow evaluations, across different ski categories, and the physical attributes of the skis. The models found can then be used to better understand the ski-snow-skier interactions

Other sectors, like the food and automotive industries, have demonstrated that complex relationships can be established between the properties of a product and its subjective evaluations by consumers [6, 7]. As the perceived behaviors of a ski depend on its interaction with the snow through its shape, deformation, and the created forces [3], one could hypothesize that similar relationships could be established using the ski’s physical measurements describing its dynamics. Unfortunately, validating such a hypothesis at a large scale has historically been hindered by the lack of detailed physical measurements and quantitative ski ratings. That situation changed recently, with *SoothSki* measuring the full geometry, the mass, and the bending and torsional stiffness distributions of more than 4700 skis commercially available and publishing the results online [8, 9]. These measurements can be paired with quantitative on-snow ratings to explore the proposed relationships, as illustrated in Fig. 1. To demonstrate the potential of this approach, it was chosen to use the ratings from *Blister Review*, an online ski review website, instead of conducting new on-snow tests. The ratings from *Blister* include 300+ models, 12 categories and 36 rankings from best to worst [10], which represent thousands of on-snow hours. The breadth of models, categories and rankings illustrate the complexity of selecting skis to match skier style, preferences, terrain and snow conditions.

The two main contributions of this study are to (1) propose a novel methodology that automatically generates linear models capable of accurately predicting the performance a sporting equipment using only a few physical measurements and to (2) apply this methodology to the on-snow performance evaluation of alpine skis. To do so, the proposed methodology addresses many challenges encountered with datasets typical of sport engineering applications. These include the small number of skis in each category compared to the number of physical properties measured, the risk of overfitting, the multicollinearities, the robustness of the created models, the prediction of a rank and the explicability of the results. Section 2 presents the methods that use a combination of elastic net regression, bootstrap, and appropriate feature selection. Section 3 presents the results, including the tuning of the methodology’s parameters, the predictive performance obtained by the models created, and a detailed example of a model. Finally, the limits of the results and their applicability, along with the insights that can be drawn from the models, are discussed in Sect. 4.

## Methods

This section presents the algorithm developed to construct simple and robust predictive models for each metric based on the physical attributes of skis that minimize the error between the predicted (${{\hat{\boldsymbol {y}}}}$ and observed (***y***) on-snow performance rank for a given metric, as shown in Fig. 1. This can be formalized as the search for the vector of coefficients ${{\hat{\boldsymbol {\beta}}}}$ in the following linear equation:

1 $${\hat{\boldsymbol{y}}} = X{\hat{\boldsymbol{\beta}}}$$

2 $$\hat{\boldsymbol{y}} = \hat{\beta }_{0} + {\varvec{x}}_{1} \hat{\beta }_{1} + \cdots + {\varvec{x}}_{{\varvec{p}}} \hat{\beta }_{p}$$

where $\hat{\boldsymbol{y}}$ is the *n* × 1 predicted rank vector of a given metric for the *n* skis of its associated category, ***X*** is a *n* × (*p* + 1) matrix containing the *p* physical attributes of the skis in that category, ***x***_***i***_ is the *i*th input attribute vector, and ${\hat{\beta}}_{{i}}$ is the *i*th predicted linear coefficient.

The data in this study is such that an indefinite number of measured ski attributes can be extracted from the raw measurements. This means that multiple collinearities between the attributes can exist and that numerous unimportant attributes could be initially included in the analysis of a specific metric. Furthermore, the number of evaluated skis is comparatively low (i.e., between 12 and 32 per category), but they are evaluated on a large number of metrics (i.e., 36). Although different techniques can be used to find ${{\hat{\boldsymbol {\beta}}}}$ coefficients [11], many of these techniques can lead to models that are unstable (i.e., the coefficients can vary widely for small changes in the dataset), that overfit the data or are hard to explain.

**Fig. 2:**
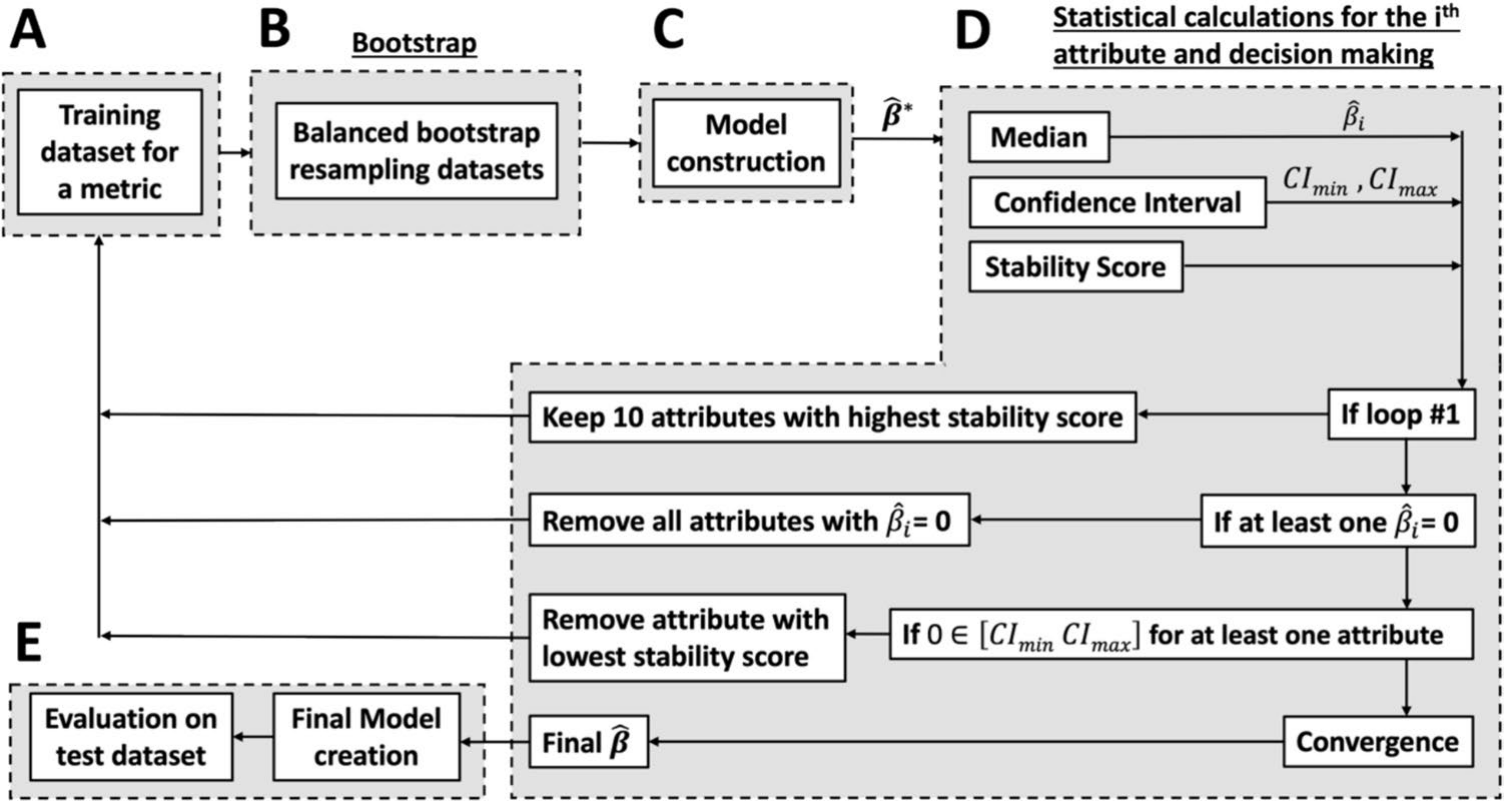
Overview of the complete algorithm used to develop the linear predictive models. $\hat{\mathbb{\beta }}^{\mathbb{*}}$ is the set of all estimated beta coefficient for each resampling (vector). The process is repeated for each metric

To address these challenges, a novel methodology was developed to combine bootstrap, Elastic Net and automated parameter selection to construct predictive linear models that are characterized by their simplicity, interpretability, and accuracy. This algorithm is presented in Fig. 2. The main steps, presented in the following subsections, are as follows: (1) the assembly of the training dataset for each metric, (2) the creation of balanced bootstrap resampling datasets, (3) the creation of models to represent the bootstrapped datasets and extract statistics on the $\hat{\boldsymbol{\beta }}$ coefficients found, (4) the iterative reduction of the attribute set through the analysis of the $\hat{\boldsymbol{\beta }}$ coefficients, their stability and their statistical significance, and (5) the evaluation and unbiased model evaluation on a test dataset upon convergence.

### Training dataset (A)

The training dataset utilized in this study consists of a combination of *Blister Reviews’* on-snow performance rankings and *SoothSki*’s physical ski measurements. The physical ski measurements use the raw measure of the full geometry, the mass and the full bending and torsional stiffness profiles on more than 4700 skis [9]. From these raw measurements, more specific attributes can be calculated (e.g., rocker length, sidecut radius, camber height, average bending stiffness, tip bending stiffness). In this study, 42 physical attributes were used as described in Online Resource 1. The attributes were normalized using the mean and standard deviation of the skis within the corresponding category of the associated subjective rankings to better represent the relative importance of the coefficient estimates.

The second part of the dataset uses subjective ski rankings obtained from the *2021–2022 Blister Winter Buyer’s Guide* [12]. These rankings were chosen as they are some of the most extensive quantitative evaluations available, both in terms of the number of skis tested and the diversity of evaluation metrics. Their testing procedure is loosely defined but typically involves testing skis over multiple days to experience a range of terrains and conditions, with comparative A/B evaluations. The on-snow tests are typically performed by one main reviewer for each category. Many of their main reviewers have been testing skis for 6+ years. All these factors contribute to the alleged consistency of their on-snow evaluations. Overall, this dataset compares a total of 261 skis, divided into 12 categories containing each 3 metrics (see Table 1 for the full list and detailed descriptions in Online Resource 1). Skis were ranked from best (#1) to worst (*n*) in the vector ***y***, with *n* corresponding to the total number of skis in each category. Each category contains between 12 and 32 skis. In this work, the ranks were normalized by the total number of skis per category to obtain a uniform 0 to 1 scaling (best to worst). To help the convergence of the algorithm and ensure the robustness of the results, only 10 of the 12 categories were used, i.e., the categories for which at least 10 skis were measured. The methodology shown in Fig. 2 was repeated 30 times, i.e., for the 3 metrics in each of the 10 categories with enough skis.

**Table 1:** Model performance for each metric (NES = Not Enough Skis, DNC = Did Not Converged)

Category	Performance Metric	Measured skis/Total skis	Nb. of retained attributes	Training dataset *R*^2^_adj_	Training dataset MAE (%)	Measured skis in test dataset	Test dataset MAE (%)	Year over year MAE (%)
50/50 Skis: Backcountry and resort	Best in variable	10/23	2	0.85	7.7	3	6.6	4.0
	Most inbounds-oriented		1	0.42	16.4		17.0	3.5
	Most playful		2	0.86	8.9		3.2	4.4
Backcountry touring skis	Best for steep, firm terrain	14/30	3	0.86	7.0	5	19.4	3.5
	Best in crusty/punchy snow		3	0.88	6.9		14.5	8.5
	Best in heavy, wet snow		3	0.81	8.5		6.7	7.4
Women’s Skis—Narrower	Best in variable	13/16	3	0.95	4.1	4	10.9	7.8
	Best on piste		3	0.70	12.9		26.0	7.1
	Best in moguls and trees		4	0.82	8.2		28.3	5.1
Women’s Skis—Wider	Best in powder	14/19	3	0.78	8.6	6	21.5	4.7
	Best in variable		2	0.66	14.5		29.3	2.8
	Best on piste		4	0.88	5.9		22.6	3.9
Frontside Skis	Most versatile	13/25	2	0.78	10.1	2	12.9	7.9
	Best in moguls		2	0.73	9.0		13.6	6.9
	Most accessible		3	0.93	5.9		13.3	4.6
All-Mountain Skis—More Stable	Best on ice	21/32	3	0.61	14.3	5	19.6	4.8
	Best suspension/damping		4	0.75	9.2		14.5	3.8
	Best in powder		4	0.86	9.1		3.3	3.0
All-Mountain Skis—More Forgiving	Best in deep snow	20/30	5	0.88	6.1	7	13.9	2.7
	Most stable in crud		4	0.83	8.2		17.5	4.1
	Most maneuverable		3	0.47	13.3		13.3	6.5
All-Mountain Skis—Chargers	Best in powder	3/12	NES	–	–	–	–	–
	Least demanding		NES	–	–		–	–
	Easiest in tight terrain		NES	–	–		–	–
All-Mountain Skis—Freestyle	Most stable in crud	13/24	1	0.74	8.6	6	9.2	5.1
	Most park-oriented		2	0.72	8.2		12.9	3.8
	Most surfy/loose		3	0.75	9.1		23.7	2.2
Park skis	Most playful	12/14	2	0.43	14.4	2	21.9	4.0
	Most stable on jumps		1	0.28	20.2		10.9	5.6
	Most versatile		3	0.82	7.9		21.1	1.6
Powder Skis—More Directional	Most flotation	7/15	NES	–	–	–	–	–
	Best in deep chop		NES	–	–		–	–
	Best in refrozen chop		NES	–	–		–	–
Powder Skis—More Playful	Most surfy/loose	10/21	DNC	–	–	2	–	–
	Lightest in the air		1	0.82	10.0		5.7	3.2
	Most stable in chop		2	0.71	12.1		1.0	5.5
Total		150/261				42		
Mean		14	2.7	0.74	9.84	4.2	14.9	4.8
Min		10	1	0.28	4.1	2	1.0	1.6
Max		21	5	0.95	20.2	7	29.3	8.5

### Creation of balanced bootstrap resampling datasets (B)

To improve the accuracy of the models and to reduce the sensitivity to small variations in the input training dataset (i.e. removing a ski, fitting the exact ranking), balanced bootstrap resampling was used. This procedure calculates the model’s coefficients $\hat{\boldsymbol{\beta }}^{\boldsymbol{*}}$ for many slightly modified datasets created by resampling the original dataset. Balancing ensures that each ski appears an equal number of times in the resampled datasets [13, 14].

**Fig. 3:**
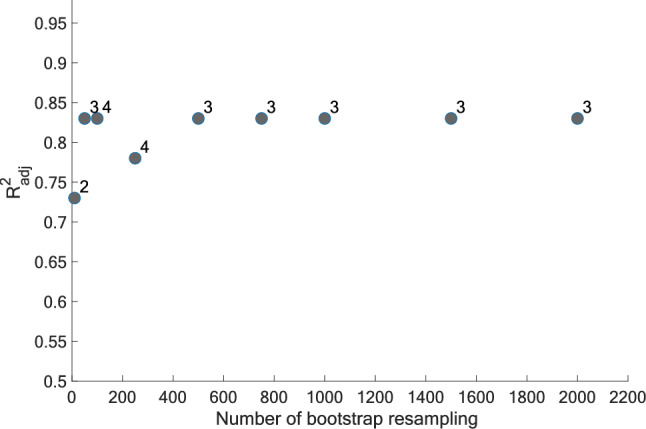
Evolution of $R_{{{\text{adj}}}}^{2}$ and the number of attributes retained in the final model for different number of bootstrap sets (NBS) for *α* = 0.9. The number of attributes is indicated next to each point

To obtain stable results, the number of bootstrapped datasets generated (NBS) needs to be selected appropriately. Up to 2000 bootstrapped datasets were considered, but no improvements in the model’s coefficient and accuracy, or in the number of retained attributes, were observed past 500 datasets during a preliminary evaluation, as shown in Fig. 3. A value of NBS = 1000 was chosen to develop all models and ensure accurate results data while limiting the computational time.

### Model construction from balanced bootstrapped datasets (C)

Block C calculates one linear model for each of the 1000 bootstrapped datasets and creates a set of linear coefficients $\hat{\boldsymbol{\beta }}^{\boldsymbol{*}}$. Many methods exist to create linear regression models when more input parameters are available than output values or when the input variables are correlated. Such methods include stepwise regression, which iteratively selects a subset of input parameters based on various criteria before developing a model [11]. Other methods, like Ridge, Lasso and Elastic Net regression, select a subset of input parameters while they develop the model [11, 15]. This is computationally faster and easier to automate. Elastic Net regression was chosen because it combines the strengths of both Lasso and Ridge regression with respect to attribute selection and grouping, stability in the presence of correlated features, and balanced coefficients obtained [16–19].

The Elastic Net algorithm requires the adjustment of two parameters: *α*, and *λ*. The parameter *α* balances the Ridge and Lasso regularization to adjust model complexity. A value of 0.9 was selected through preliminary tests, as detailed in Sect. 3.1. For a given *α*, the parameter *λ* determines the overall strength of the regularization. Higher values of *λ* result in more shrinkage, leading to smaller coefficient magnitudes and increased model simplicity. On the other hand, lower values would require more time to reduce the attribute set and converge to a simple model. The appropriate value was selected automatically using cross-validation (CV) to obtain a minimal mean square error on the training dataset [7, 16]. A 5 k-fold CV was selected to keep a minimum amount of skis in the validation set. However, the exact number of folds had limited influence on the results. The Elastic Net algorithm was implemented through the *lasso* function in *MATLAB® 2022a* [20].

### Model reduction through stability and statistical significance improvement (D)

Block D aims at simplifying the final model by iteratively reducing the attribute set available to build the models in block C, while also minimizing the potential loss of accuracy or eliminating attributes too quickly. To do so, the algorithm follows three steps. The first step looks at the stability score of each attribute, as proposed in references [19, 21], to select 10 attributes from the full list. The stability score measures how frequently a particular attribute is retained in all the models created through the bootstrapped datasets in block C. Attributes that are consistently retained across multiple datasets are considered more stable and are more likely to be truly important attributes. The selection of the top 10 attributes is motivated by the desire to rapidly decrease complexity while preventing the removal of important attributes too early, as seen in Fig. 5. Throughout the iterations, as a reduced set of attributes is used, higher stability scores are obtained with more precise and reliable coefficient estimates.

To further reduce the model, attributes are eliminated when the median value of their associated coefficient is zero. This is associated with attributes that have not been retained by the Elastic Net algorithm most of the time. When no more coefficients can be eliminated through this step, the methodology evaluates the statistical significance of the remaining attributes. When one or more attributes are deemed non-significant the attribute with the lowest stability score is eliminated. Statistical significance is assumed when the 95% confidence interval (CI) of a coefficient does not encompass zero [13], meaning that the bootstrapped models agree in the way that each attribute influences the outcome. The confidence interval is calculated with the 2.5th and 97.5th percentiles of the coefficient found with the bootstrapped models.

The algorithm progresses to block E after full convergence, which signifies that all retained attributes possess non-zero coefficients and are statistically significant.

### Final model creation and unbiased performance evaluation (E)

Once the algorithm converges to a reduced set of attributes for a given metric, the final linear model is built from all the 2021–22 skis available for that same metric. Given the variable number of attributes retained for different metrics, the adjusted coefficient of determination ($R_{{{\text{adj}}}}^{2}$) is used to evaluate the predictive performance on the training dataset [22].

An unbiased evaluation of the final models is performed using an independent test dataset of the skis from the following season, i.e., the *2022–23 Blister Winter Buyer’s Guide* [23]. To prevent the inclusion of skis in both the training and test datasets, unchanged skis between the 2021–22 and 2022–23 seasons were excluded from the test dataset. This information is specified by *Blister* and confirmed with the measurements. This left 93 skis in the test dataset out of a total of 270 skis in 2022–23. Prior to prediction, the 2022–23 data were normalized using the mean and standard deviations found from the 2021–22 categories.

To evaluate the models’ performance on both the training and test datasets, the Mean Absolute Error (MAE) is used. The MAE quantifies the mean rank error between predicted and actual values. This metric was chosen over the coefficient of determination due to the relatively small number of skis available in the test dataset, and because it better represents the accuracy of the rank prediction when compared to other statistics based on rank correlation (e.g., Spearman or Kendall rank correlation coefficient). The MAE between the 2022–23 and 2021–22 rankings was also calculated using the 2021–22 skis returning unchanged in 22–23. This serves as an indication of the stability of the ranks from one year to the other.

## Results

This section presents the results of the parameter tuning and its influence on the attribute selection process. It then summarizes the performance of all the final models and describes, as an example, one specific metric in more detail.

### Parameters tuning and attributes selection process

To identify the number of bootstrapped sets (NBS) needed to produce stable models, the NBS was varied while the $R_{{{\text{adj}}}}^{2}$ of the final model and the number of attributes retained was monitored. Figure 3 shows an example for the category *All-Mountain More Playful* and the metric *Most Stable in Crud*. It is possible to observe that $R_{{{\text{adj}}}}^{2}$ remains constant with more than 500 bootstrap resamples and that a constant number of attributes was also retained. As described before, the NBS was set to 1000 for all metrics to provide an additional safety margin while keeping a reasonable computational cost.

**Fig. 4:**
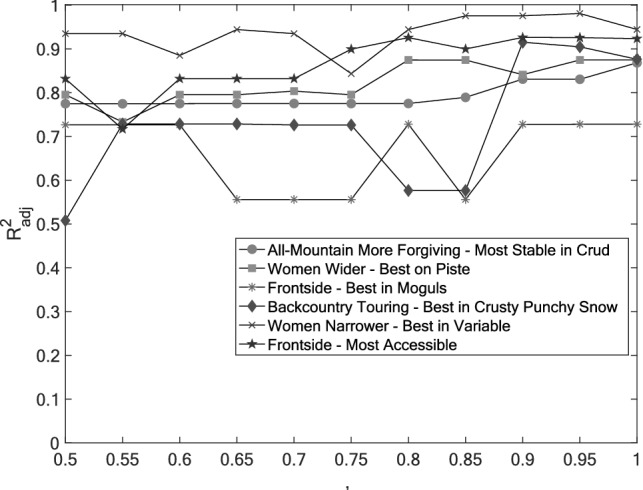
Adjusted *R*^2^ values for six distinct performance metrics as *α* varies from 0.5 to 1

As detailed before, the value of *α* in the Elastic Net algorithm controls the regularization used and, effectively, how many attributes are retained in each loop of the algorithm. For example, using a value of 0.5 would typically result in more than 30 of the 42 attributes remaining after the initial loop, while using a value of 0.9 would typically retain only 10 attributes. Selecting an appropriate value is required for both accuracy and speed of convergence. Figure 4 presents the impact of *α* on the overall performance for a few metrics of the dataset. It is possible to observe that the proposed methodology is relatively robust to the choice of *α*, even if a value between 0.9 and 0.95 yields the best fit. For consistency throughout the study, an *α* of 0.9 was selected for all performance metrics presented next.

**Fig. 5:**
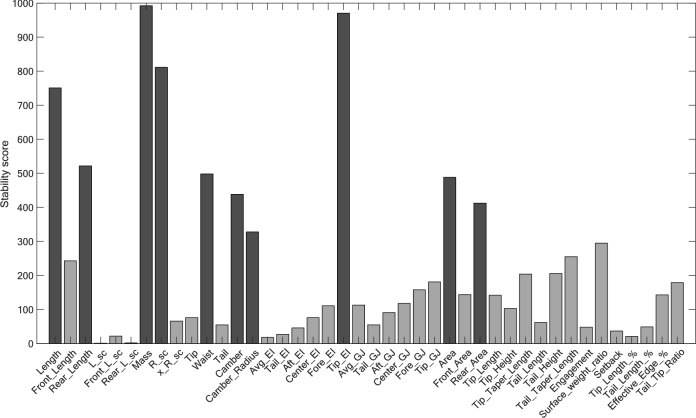
Stability score for each physical attribute during the first iteration of the proposed methodology for the metric “Most Stable in Crud” in the category “All-Mountain More Forgiving”

As detailed in paragraphs 2.3 and 2.4, ensuring appropriate attribute selection is essential to obtain predictive models that demonstrate good performance while remaining simple and using statistically significant attributes. Under noisy input and outputs, the retained attributes may vary across the models created for each bootstrap resampling. Figure 5 presents the stability score for each attribute during the initial pass-through of block D. As the stability score quantifies how frequently a particular attribute is used across the 1000 bootstrapped sets, it thereby sheds light on attributes that are most likely to be truly important. For instance, Fig. 5 highlights that *Mass* and *Tip_EI* were consistently present in almost all resampling models of the metric *Most Stable in Crud* of the category *All-Mountain More Forgiving*, while *Waist* was retained approximately half the time. The 10 attributes having the highest stability score, highlighted in dark grey, are retained for the next iteration of the algorithm. However, it is essential to recognize that a high stability score does not guarantee statistical significance. For example, in the first loop, *Tip_EI* had the second-highest stability score but was eventually removed in the sixth iteration due to its confidence interval crossing zero.

### Created models

Upon completion of the iterative algorithm, signifying the convergence of the attribute selection and their statistical significance, the final model for the metric under consideration is constructed. The results for all the metrics are outlined in Table 1. These results encompass the $R_{{{\text{adj}}}}^{2}$ values obtained on the training dataset, the Mean Average Error (MAE) assessed on the training and the test dataset, and the MAE between 2022–23 and 2021–22 on-snow rankings. The models used on average 2.7 attributes to predict the rankings. The average $R_{{{\text{adj}}}}^{2}$ obtained was 0.74, with some models reaching 0.95. The MAE was, on average, 9.8% on the training dataset and 14.9% on the test dataset. The MAE of the rankings from one year to the other was 4.8%.

**Fig. 6:**
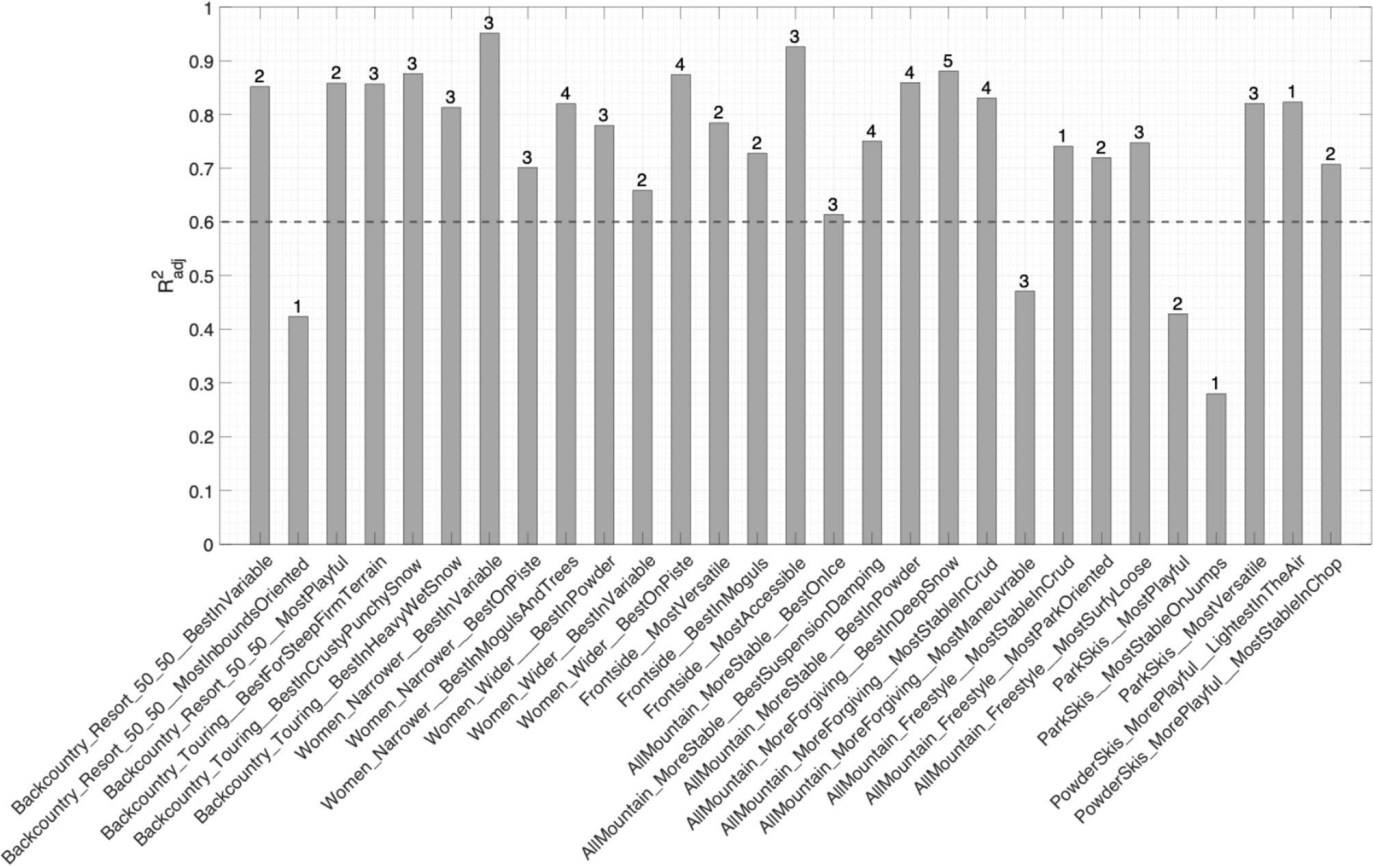
$R_{{{\text{adj}}}}^{2}$ for all performance metrics (*α* = 0.9). The number of retained attributes is shown on top of each vertical bar

More specifically, Fig. 6 shows the $R_{{{\text{adj}}}}^{2}$ values on the training dataset for each metric along with the number of retained attributes. Of the 30 metrics having 10 or more skis in their category, 25 had a $R_{{{\text{adj}}}}^{2}$ greater than 0.6 and only 1 did not converge.

**Fig. 7:**
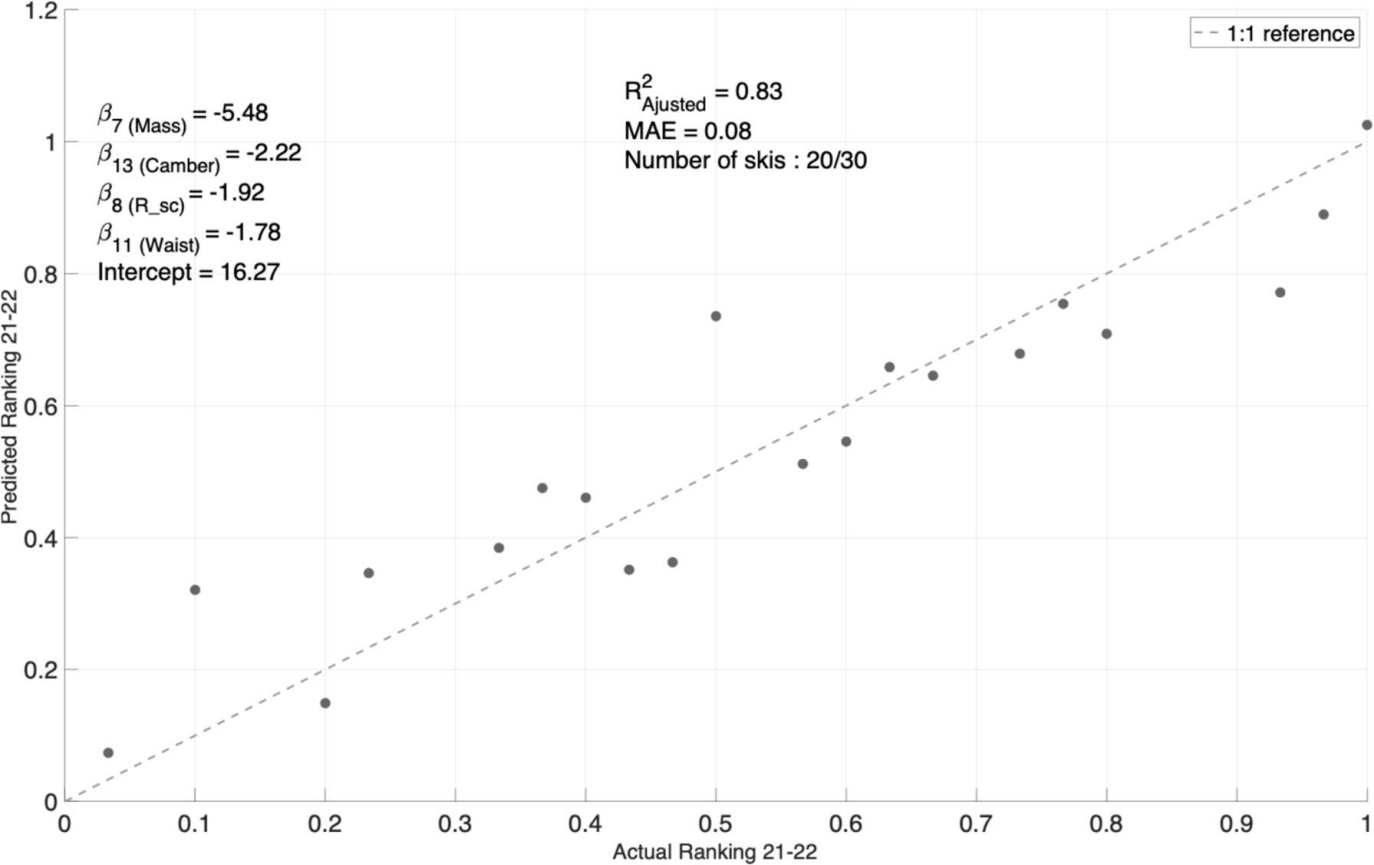
Predicted “Most stable in crud” ranking against the actual on-snow ranking for the “All-Mountain More Forgiving” category (training dataset). Retained attribute coefficients are listed in the top left

Figure 7 shows a specific example of the ranking predicted by the final model for the metric *Most Stable in Crud* in the *All-Mountain More Forgiving* category when applied to the 2021–22 dataset. Each point represents an actual-predicted ranking combination, while the dotted line represents a perfect prediction (1:1 reference). The model achieves $R_{{{\text{adj}}}}^{2}$ of 0.83, utilizing only four attributes: *Mass*, *Camber*, *Sidecut Radius (R_sc)*, and *Waist*. With this concise set of attributes, the model predicts the rankings of the 20 measured skis with an MAE of 8%, highlighting its accuracy in performance prediction on the training dataset.

**Fig. 8:**
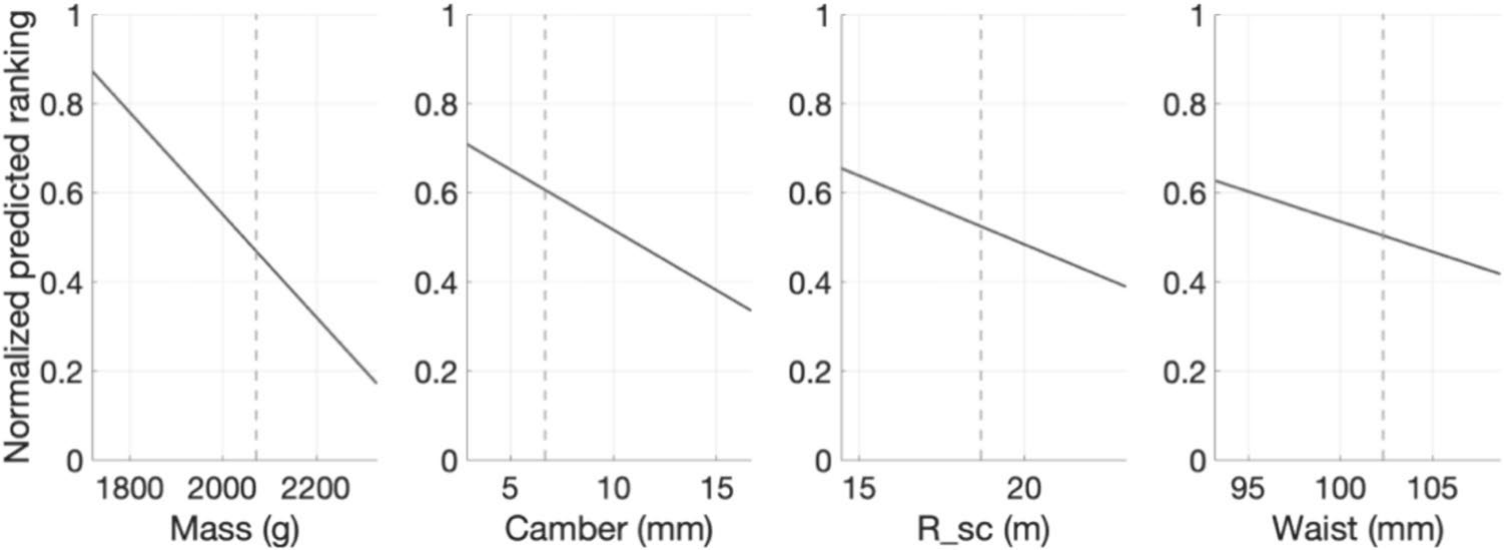
Prediction slice plots showing the variation in predicted ranking for given variation in the physical attributes, for the “Most Stable in Crud” metric in the “All-Mountain More Forgiving” category. Lower rank values are better. The horizontal axes are scaled from the attribute spread seen in that category, while the dotted line corresponds to the mean of each attribute

In the models found, since the input attributes were normalized, it is possible to easily compare the relative importance of each attribute retained. For example, using the coefficients listed in Fig. 7, it is possible to notice that the mass influence on the *Most stable in crud* metrics is 2–3 times greater than the other three attributes. Figure 8 visually demonstrates the variation in the ranking score for each corresponding absolute variation in the physical attributes retained.

**Fig. 9:**
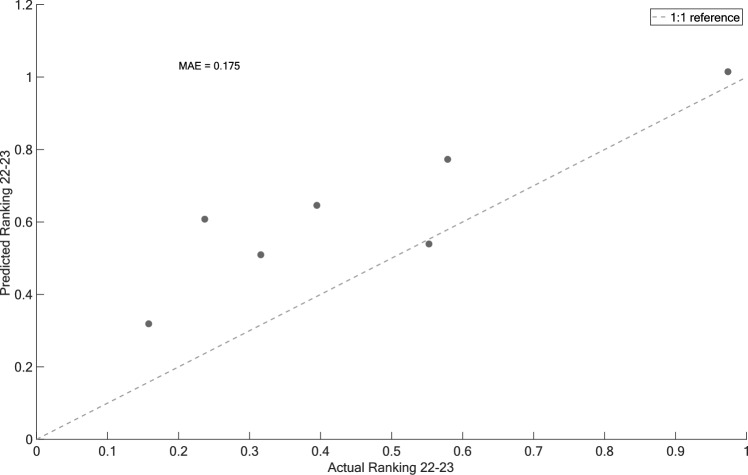
Predicted 2022–23 rankings for “Most stable in crud” metric versus the actual on-snow rankings in the “All-Mountain More Forgiving” category (test dataset)

Figure 9 shows the predictions of the 2022–23 rankings from the model developed with the 2021–22 dataset. In this category, *All-Mountain More Forgiving*, the 2022–2023 dataset contained seven measured skis. The model built using the 2021–2022 ski data predicted the 2022–2023 ranking with a MAE of 17.5%.

## Discussion

The main aim of this work was to develop a methodology that automatically generates robust and explainable models of the on-snow performance of alpine skis based on their measurable physical characteristics while addressing challenges typically encountered in sports equipment research (e.g., a small number of models in each category, a high number of physical attributes measured, potential for overfitting, multicollinearities). The proposed methodology converged for 29 of the 30 metrics attempted in Table 1. Of the metrics that converged, 25 reproduced the on-snow ski rankings with an $R_{{{\text{adj}}}}^{2}$ greater than 0.6. Furthermore, the mean average error (MAE) of the predicted rank on the training dataset is, on average, 9.8%, and under 15% for all metrics except for two. The average MAE on the test dataset, i.e., for skis never seen before by the algorithm, increases to only 15%. This confirms the robustness of the models and the ability of the methodology to retain appropriate attributes and avoid overfitting. In comparison, the average MAE of the 22–23 rankings given by the on-snow reviewers when compared to the 21–22 rankings is 4.5%, with a maximum MAE of 8.5%. This gives an idea of the year-over-year variability of the reviewer-based on-snow rankings and an upper bound on the performance expected from a predictive model built on these results. The MAEs obtained on the test dataset suggest that the models obtained could confidently predict a ski’s performance on a scale with 3 to 5 levels.

It is noteworthy that most models achieved convergence and an MAE below 15% with only 10 to 21 skis per category, particularly given the complexity of the ski-snow-skier interactions they aim to characterize. Interestingly, the relationship between the Training Dataset MAEs and the number of skis in the associated training category is almost flat, with an *R*^2^ of 0.009. This suggests that adding more skis would not improve the results. Many factors could explain this result, starting with the use of cross-correlation and bootstrap in the proposed methodology. This creates, during the model development, several subsets of the total number of skis available and retains only the most common features of the models created. Another explanation is that the chosen metrics represent the most noticeable on-snow feels for a given category. Differentiating more subtle differences between skis might require adjustments to the model development methodology, the on-snow tests procedure and the number of skis used. Finally, a total of twelve relatively narrow categories are used to first classify the skis in the dataset, creating a local approximation of each metric. A dataset with fewer categories might require more complex attribute descriptions (e.g., deformed shapes) and non-linear predictive models. This might explain the higher MAE observed in the *Women* categories that include a higher diversity of skis (i.e., only two categories compared to ten different categories for the unisex skis).

The predictive power of the models suggests that both (1) the physical attributes measured on these skis are sufficient to describe the primary behavior perceived during the on-snow tests and that (2) the on-snow evaluations were sufficiently consistent within and across different years. This is a significant finding, as ski design is often considered challenging to quantify, particularly when certain attributes were historically neither accounted for nor controlled (e.g., torsional stiffness, bending and torsional stiffness distributions). While some physical attributes, such as the mass distribution and damping, may still require direct measurement to further improve the results, it is notable that the *Best Suspension/Damping* metric could be predicted with a MAE of only 14.5% despite the absence of direct damping measurements (which is typically small, and dependant on the snow and boundary conditions [24, 25]). In this case, ski mass alone accounts for more than 68% of the ranking variance, either directly or through correlated effects (e.g., the inclusion of metal/rubber layers or variations in wood species). The predictive performance of most metrics further underscores the reliability of the on-snow evaluations, which are often considered highly subjective. These findings demonstrate that physically consistent on-snow evaluations can be achieved at scale, particularly when conducted by a small team of trained evaluators who systematically identified the most distinguishing metrics for each category.

Although models were developed successfully, suggesting consistent on-snow evaluations, reproducing the on-snow rankings used in this work would be challenging given the current definitions of categories and metrics. In addition to the lack of clear definitions in some cases, ski classification often relies on either its on-snow feel (e.g., “more stable”) or a specific physical attribute (e.g., “generally stiffer”), necessitating on-snow testing before a ski can be assigned to a category. Similarly, the metrics used for ranking typically reference either a behavioral attribute (e.g., versatile, accessible, maneuverable, demanding), a terrain type (e.g., steep, piste) or a snow condition (e.g., variable, crusty, powder, ice). To enhance clarity and reproducibility, these aspects should be explicitly defined for all metrics. Additional relevant factors, such as turn type, tester skill level, skiing style, could also be incorporated. Furthermore, metrics labelled as “Best in …” typically represent a general assessment under specific conditions without explicitly defining the characteristic being evaluated. For instance, in the case of “Best in moguls”, different rebound characteristics might be preferred depending on the skier’s skill level, with beginners favoring a forgiving ski while competitive racers may seek one that efficiently returns energy to facilitate acceleration toward the finish line. More broadly, it is important to point out that the field would benefit from better defined on-snow testing procedure. For example, the ISO 8783:2015 norm remains largely unchanged since its introduction in 1985 [5], predating modern ski technologies like shaped sidecut, powder-specific skis and lightweight touring constructions [26]. As such, it limits testing to hard snow and defines only six vaguely specified evaluation metrics, omitting key best practices in sensory testing needed for inter- and intra-rater reliability [27]. Consequently, the standard has seen little adoption in research and industry.

Despite these challenges in defining categories and metrics, the resulting models remain simple and highly interpretable, offering valuable insights into the metrics considered. Indeed, of the 29 models that converged, four of them use one attribute, 19 use between two and three attributes, and six use between four and five attributes. Along with the obtained MEAs, this highlights the algorithm’s ability to achieve good predictive performance while utilizing a relatively small number of attributes. This could enable designers to develop a deeper understanding of the physical principles involved in each metric. For example, Figs. 7 and 8 show that four attributes, namely mass, turning radius, camber height, and waist width, are important in establishing the ranking of an *All-Mountain More Playful* ski with respect to its stability in crud snow (i.e., *heavy, grabby and often hard clumps of chopped up snow* as described by reference [28]). The results make physical sense, as one can easily imagine that a heavier ski will “punch through” that snow, that a longer turning radius will make the tip and tail less “catchy”, that more camber will pressurize the tip and tail to prevent them from “vibrating” at high speed in rough terrain, and that a larger waist width will allow the skier to “float” better over uneven snow. The models also quantify how much each property affects the rankings. For example, the model illustrated in Figs. 7 and 8 suggests that a 150 g increase in the ski’s mass is equivalent to a 7 mm increase in camber. These results could be used to better understand how to improve a given metric or how to improve a metric without affecting other important characteristics of a ski.

The physical attributes retained by a model are the ones providing the best fit for the skis in that category. Some important attributes might thus not be present in the models found by the algorithm because they are already implied by their inclusion in a given category. For example, *Frontside* skis are typically much shorter than *Powder* skis (i.e., the mean lengths are, respectively, 178.6 and 182.9 cm), even if the length of the skis is not necessarily included in the models associated with these categories. Similarly, the skis selection in each category might hide some important parameters (e.g., skis are typically selected in lengths most preferred by the main reviewer). One could deal with these challenges by considering the physical attributes involved in defining each category like in reference [29], by including more skis within a category, or by developing datasets without categories.

**Fig. 10:**
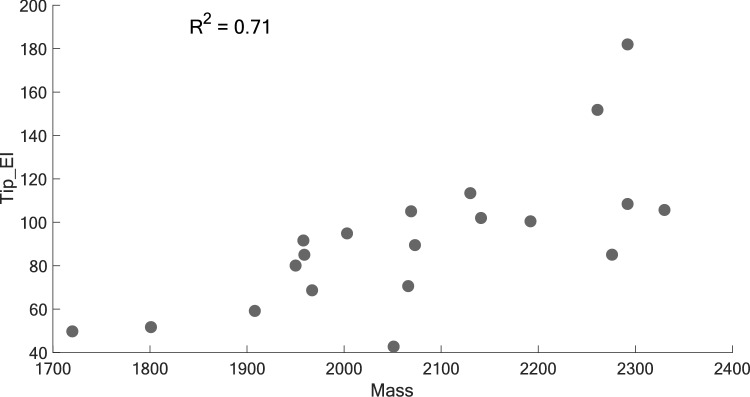
Correlation between Mass and Tip_EI

To gain a deeper understanding of the important properties for a given metric, it is possible to look at the attributes most correlated with the attributes retained in the model as proposed in reference [29]. For instance, Fig. 10 reveals a moderate correlation between a ski’s mass and its tip bending stiffness (*R*^2^ = 0.71). This correlation is expected given that the bending stiffness is often, but not always, increased by adding more layers of structural materials and by thickening the wood core of the ski. That being said, replacing the *Mass* attribute by *Tip_EI* in the model changes the $R_{{{\text{adj}}}}^{2}$ from 0.83 to 0.61. Unfortunately, only a specifically collected dataset could confirm the exact role of each correlated parameter by varying these attributes independently, potentially requiring much more time and effort (e.g., using custom prototypes and more systematic on-snow testing).

**Fig. 11:**
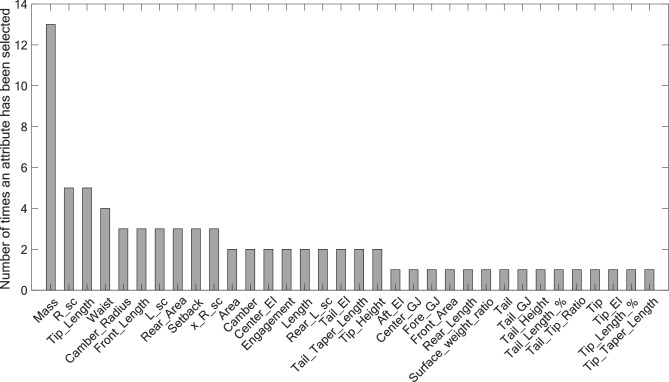
Distribution of attribute occurrences in the 29 developed models

Interestingly, a subset of attributes emerges as significant contributors to the perceived on-snow performance as they are consistently retained across multiple metrics. The distribution of attribute occurrences is depicted in Fig. 11. Attributes such as mass, sidecut radius (*R_sc*), front rocker (*Tip_Length*), and waist width are retained by the algorithm 76% of the time. This might be explained by the fact that many metrics are based on how the skis interact with different types of soft snow (e.g., chop, powder, deep powder, crud, crusty, wet). Consequently, when selecting or designing such skis, due consideration should be given to these attributes. Metrics associated specifically with the bending and torsional stiffnesses of the skis (i.e., EI and GJ) are *Best in Variable* (Women Narrower), *Best in Moguls and Trees* (Women Narrower), *Most Maneuverable* (All-Mountain More Forgiving), *Most Surfy/Loose* (All-Mountain Freestyle), *Most Playful* (Park Skis), *Most Versatile* (Park Skis), *Best on Ice* (All-Mountain More Stable), *Best in Powder* (All Mountain More Stable) and *Most Playful* (Backcountry-Resort 50/50). Other attributes might play a finer role in the metrics analyzed, be important for only a few metrics, be correlated to other metrics or already be implied by the chosen categories. These reasons could also explain why many of the retained attributes are geometrical quantities (e.g., *Chargers* skis might be generally stiffer in bending and *Forgiving* might be softer in torsion). Further works could also consider including more complex attributes calculated from the measured mechanical properties. One example is the pressure distribution along the edge during a turn [30, 31], which combines the geometrical and mechanical ski properties, but also snow properties and skier’s loading. This could add the benefit of including the skiers’ and snow effects during the testing.

## Conclusion

Through the synergy of the *SoothSki* database, featuring measurements of commercially available skis, and the on-snow evaluation rankings of *Blister Gear Review*, a new opportunity emerged to develop a large number of predictive models providing valuable insights into the relationship between a ski’s mechanical properties and its on-snow performance. The presented methodology combines Elastic Net regression with balanced bootstrap resampling and feature selection to create accurate and stable predictive models automatically. The models exhibited an $R_{{{\text{adj}}}}^{2}$ on the training dataset larger than 0.60 for 25 of the 30 metrics and have a mean rank prediction MAE on the test dataset of 15%. Furthermore, the models utilize less than three physical attributes in most cases to make their prediction. These results suggest good predictive capabilities and low overfitting with simple models that can offer insight into the most important physical properties that define a ski’s performance. The results are also, to the authors’ knowledge, the most comprehensive and extensive description of alpine skis’ on-snow performance. The methodology presented and the predictive models developed hold potential utility for the ski industry, such as in streamlining the design process and guiding skiers in their purchase decisions. While the work presented has focused on utilizing a specific set of on-snow evaluations, the automated methodology could be readily adapted to accommodate other sources of on-snow evaluations. This would offer opportunities for further model refinements and improvements, and the comparison of diverse opinions about the on-snow feel of alpine skis.

## Supplementary Information

Below is the link to the electronic supplementary material.

Supplementary file1 (DOCX 26 KB)

## Data Availability

The data that support the findings of this study are available from the corresponding author upon reasonable request.
