# Supplementary material for: Insightful skiing: developing explainable models of on-snow performance through physical attribute selection of alpine skis
Source: Sports Eng. 2025 Aug 4;28(2):35. doi: 10.1007/s12283-025-00511-w (PMC12327194; doi:10.1007/s12283-025-00511-w)
Supplement: Supplementary file 1 — Supplementary file1 (DOCX 26 KB) [file 12283_2025_511_MOESM1_ESM.docx]

**ONLINE RESOURCE 1**

This section presents a brief description of the list of attributes used in this research.

# Key Terms - Attributes

| **#** | **Attributes** | **Unit** | **Description** |
| --- | --- | --- | --- |
| **1** | Length | cm | Total length of the ski |
| **2** | Front_Length | cm | Length from tip to boot center |
| **3** | Rear_Length | cm | Length from tail to boot center |
| **4** | L_SC | cm | Longitudinal length between widest tip point and widest tail point |
| **5** | Front_L_SC | cm | Longitudinal length between widest tip point and boot center |
| **6** | Rear_L_SC | cm | Longitudinal length between widest tail point and boot center |
| **7** | Mass | g | Mass of a single ski |
| **8** | R_SC | m | Sidecut radius |
| **9** | x_R_SC | cm | Longitudinal length between radius center and boot center |
| **10** | Tip | mm | Widest ski tip width |
| **11** | Waist | mm | Minimum width of the ski around boot center |
| **12** | Tail | mm | Widest ski tail width |
| **13** | Camber | mm | Highest distance between ski and ground at its natural shape near boot center |
| **14** | Camber_radius | m | Radius of the camber at its natural shape near boot center |
| **15** | Avj_EI | Nm^2^ | Mean bending stiffness |
| **16** | Tail_EI | Nm^2^ | Mean bending stiffness of the 1st/5 ski length part from tail |
| **17** | Aft_EI | Nm^2^ | Mean bending stiffness of the 2nd/5 ski length part from tail |
| **18** | Center_EI | Nm^2^ | Mean bending stiffness of the 3rd/5 ski length part from tail |
| **19** | Fore_EI | Nm^2^ | Mean bending stiffness of the 4th/5 ski length part from tail |
| **20** | Tip_EI | Nm^2^ | Mean bending stiffness of the 5th/5 ski length part from tail |
| **21** | Avj_GJ | Nm^2^ | Mean torsional stiffness |
| **22** | Tail_Gj | Nm^2^ | Mean torsional stiffness of the 1st/5 ski length part from tail |
| **23** | Aft_GJ | Nm^2^ | Mean torsional stiffness of the 2nd/5 ski length part from tail |
| **24** | Center_GJ | Nm^2^ | Mean torsional stiffness of the 3rd/5 ski length part from tail |
| **25** | Fore_GJ | Nm^2^ | Mean torsional stiffness of the 4th/5 ski length part from tail |
| **26** | Tip_GJ | Nm^2^ | Mean torsional stiffness of the 5th/5 ski length part from tail |
| **27** | Area | cm^2^ | Surface area of the ski when viewed from directly above or below |
| **28** | Front_Area | cm^2^ | Surface area from tip to boot center |
| **29** | Rear_Area | cm^2^ | Surface area from tail to boot center |
| **30** | Tip_Length | cm | Longitudinal length between ski tip end and tip touching the ground |
| **31** | Tip_Height | mm | Maximum height of the ski tip from the ground |
| **32** | Tip_Taper_Length | cm | Longitudinal length between ski tip end and widest tip point |
| **33** | Tail_Length | cm | Longitudinal length between ski tail end and tail touching the ground |
| **34** | Tail_Height | mm | Maximum height of the ski tail from the ground |
| **35** | Tail_Taper_Length | cm | Longitudinal length between ski tail end and widest tail point |
| **36** | Engagement | cm | Longitudinal length between tip snow contact point and widest tip point |
| **37** | Surface_weight_ratio | cm^2^/g | Surface to Mass Ratio |
| **38** | Setback | cm | Length from true center to boot center |
| **39** | Tip_Length_% | - | Normalized Tip_Length with ski length |
| **40** | Tail_Length_% | - | Normalized Tail_Length with ski length |
| **41** | Effective_Edge_% | - | Normalized L_SC with ski length |
| **42** | Tail_Tip_Ratio | - | Ratio of tail to tip height |

**Key Terms – Ski categories**

*Blister Reviews* provides a short overview of each category in its Gear Guide. The most important parts of these descriptions are listed below to provide more context [12]. Please refer to the paper for a discussion on the limitations of these descriptions.

| **#** | **Categories** | **Description** |
| --- | --- | --- |
| **1** | 50/50: Backcountry & Resort Skis | *“If you intend to use a single ski setup for both lift-accessed skiing and backcountry touring.* *The skis in this section perform “fairly well” to “quite well” across the typical range of resort conditions better, on the whole, than the generally lighter skis in our dedicated “Backcountry Touring Skis” section, while not being as heavy as many of the skis in our “All-Mountain” and “Powder Skis” sections (though these 50/50 skis are often less damp and stable).”* |
| **2** | Backcountry Touring Skis | *“Choosing a touring setup is all about finding the right combination of uphill performance and downhill performance for you and your backcountry objectives.”* |
| **3** | Women’s Skis - Narrower | *“The skis in this “Narrower” women’s section are generally best suited to firmer and/or shallower snow conditions than those in our “Wider” women’s section, though there are several in this section that still perform well in fresh snow.”* |
| **4** | Women’s Skis - Wider | *“The skis in this “Wider” women’s section generally perform better in softer and/or deeper conditions than those in our “Narrower” women’s section, though many of the skis in this section still carve very well and do not need fresh snow to be enjoyable.”* |
| **5** | Frontside Skis | *“So we’ll use the term “Frontside” to encompass three different groups of skis: (1) dedicated carvers that will only be used on groomed runs; (2) skis that carve pretty well and also work pretty well in moguls; and (3) skis that will be used primarily on groomers and in moguls, but that will also spend a little bit of time in broader off-piste terrain (e.g., “frontside” runs that include sections of steeps, trees, etc).”* |
| **6** | All-Mountain Skis – More Stable | *“The skis in this section are generally stiffer and less tolerant of mistakes (especially tail gunning, i.e., trying to ski or steer from the tails rather than the front of the ski) than those in our “More Forgiving” group. But the skis here tend to be more damp and / or stable at speed, which makes many of them easier to ski fast through tracked-out snow, variable conditions, and roughed-up groomers.”* |
| **7** | All-Mountain Skis – More Forgiving | *“This section is made up of a very diverse group of directional skis, but what they all have in common is that they (1) work well at slower speeds, (2) won’t immediately punish backseat skiing, and (3) require less input and power than many of the skis in our “All-Mountain Chargers” and “All Mountain - More Stable” sections. The skis in those sections work best when skied fast and/or aggressively, while the skis here are relatively accessible, intuitive, and easier to turn at slower speeds.”* |
| **8** | All-Mountain Chargers | *“So if you prioritize high-speed stability over low-speed maneuverability, and if you love it when your skis stay calm and composed when you’re going very fast, then welcome.”* |
| **9** | All-Mountain Freestyle Skis | *“Compared to our other “All-Mountain” sections, the skis here are generally more forgiving, more maneuverable, and better suited to skiers who like to spin, flip, ski switch, and otherwise take a more playful approach to the whole mountain.”* |
| **10** | Park Skis | *“Those who want stiff, stable skis for the biggest jumps they can find still have some good choices that have proven their capabilities over many years. But those who like to play around at slower speeds, on smaller features, and who generally prefer to take a more creative approach have more options than ever before.”* |
| **11** | Powder Skis – More Directional | *“Every fat ski that has a pretty traditional mount point (i.e., ~ 9 cm behind center). But these days it’s really not as simple as that. The skis in this section are not necessarily heavier, or far more stable, or much straighter (have less sidecut) than the skis in our “More Playful” pow skis section. But in general, the skis here will work well for those who ski with a more traditional, forward stance, and who aren’t looking to spin or ski switch.”* |
| **12** | Powder Skis – More Playful | *“In general, the skis in this section are less about driving your shovels in pow (as if you were carving up a groomer on a skinny ski), and more about surfing, smearing, spinning, and / or tricking your way through deep snow with more of an upright, centered stance.”* |

**Key Terms – Ranking metrics**

*Blister Reviews* does not clearly define the *Spectrums* used to rank the skis or their testing procedure*.* However, their website includes a glossary of some of the terms used. The table below is an extract of the terms related to the *Spectrums*, or of terms closely related when the exact definition cannot be found [24]. Please refer to the paper for a discussion on the limitations of these descriptions.

| **#** | **Metric** | **Description** |
| --- | --- | --- |
| **1** | Variable | *“An inconsistent mix of firm and soft snow. Could be a firm, maybe wind-scoured base with some looser soft snow sitting on top, or could be a mix of chunky, icy stuff and softer, gloppy snow. One of the more challenging conditions to ski fast in, and a great test of a ski’s stability.”* |
| **2** | Inbounds-oriented | Definition not found. |
| **3** | Playful | *“Can refer to both a skiing style and how a ski feels on snow. In terms of skiing style, “playful” is a more general term than “jib” or “freestyle;” you can ski playfully without throwing tricks, hitting rails, flipping, etc. Someone who skis playfully tends to ski with more of an active / dynamic style (see below), often slides and slashes their turns, likes to get in the air often, and may throw tricks and/or ski switch. In terms of a ski being “playful,” we use that word to describe skis that are (1) easy to release from a turn / slarve, (2) that produce energy when you flex them, (3) are easy to flick around in the air, (4) feel balanced in the air (often due to a more forward, centered mount point), and/or (5) ski switch well. Many skis are playful in some of those aspects, but not in others, which is why we often expand on a given ski’s “playfulness” and detail in which specific ways it feels playful.”* |
| **4** | Steep, firm terrain | *“Firm is a more general description that could refer to a cold groomer that has been skied a bit and erased of its groomed corduroy tracks, or off-piste / ungroomed conditions that are pretty hard but consistent in their smooth texture. These conditions will test the limits of a ski’s edge hold.”* |
| **5** | Crusty/Punchy snow | *“Snow somewhat similar to breakable crust, but with a less consolidated surface layer.”* |
| **6** | Heavy, wet snow | Definition not found. |
| **7** | On piste | *“Runs that have been cleared of trees and groomed (typically by a snowcat) to create an open run with smooth, compacted, consistent snow. Depending on temperature, humidity, time of day, and the skill of a mountain’s grooming staff & machinery, the surface conditions of groomers can vary quite a bit, but the main point is to have a consistent surface on which to carve nice turns. “Piste” is the French term that generally refers to the same concept, with “off piste” referring to the terrain and conditions on a mountain that are not groomed.”* |
| **8** | Moguls and trees | *“Moguls: Mounds of snow with troughs in between that form in off-piste / ungroomed terrain because of skiers making lots of shorter, skidded turns. These can vary a lot in terms of size, spacing, and snow conditions. Moguls that are widely spaced with shallow troughs and broad mounds are much easier to navigate than tightly spaced ones with deep troughs and giant, car-sized mounds.”* |
| **9** | Powder | *“Freshly fallen snow, and the thing that many skiers spend their entire lives seeking. Powder can vary a lot in terms of consistency, which often comes down to wind and temperature. In warmer climates, powder is often denser, wetter, and/or heavier, while colder temps lead to “blower” or “champagne” powder that is extremely low-density and makes for pretty effortless skiing as the snow itself provides very little resistance when heading down a slope. Wind will move fresh powder and wind-deposited snow is often denser than freshly fallen snow.”* |
| **10** | Versatile | Definition not found. |
| **11** | Accessible | *“This term is closely related to “forgiving” and “engaging.” When we say a ski is accessible, we typically mean that it doesn’t require excellent technique, lots of physical effort, or high speeds to be enjoyable to ski. Skis that work well for beginners and intermediates are often pretty accessible, but can be enjoyed by skiers of all skill levels, depending on their personal preferences.”* |
| **12** | Ice | *“Groomers:Bulletproof: Rock-hard Ice. Good luck getting your ski edges to dig in and bite. If you’re not on race or at least well-tuned piste-specific skis, the best you can probably hope for is a predictable, controlled skid.”* |
| **13** | Suspension/Damping | *“Suspension: This is a term we use a lot, and while it’s not something you’ll see in all ski reviews or descriptions, we think it’s an extremely important aspect of on-snow performance. In short, we use it similarly to “damping,” but we think “suspension” goes a bit further in terms of describing a ski’s ride quality. When we talk about a ski’s suspension, we’re talking about how it deals with and reacts to impacts and vibrations from the snow. Skis with excellent suspension do a really good job of absorbing and managing harsh vibrations from (typically firm and/or rough, bumpy) snow. Those skis are typically heavy. Skis that offer poor suspension will transmit a lot of feedback from the snow to your body, resulting in a harsher-feeling ride quality and a generally less stable, composed, and pleasant experience on firm, rough snow.”*  *Damping: “Not relating to how wet something is, but instead refers to how well a ski “mutes out” or decreases vibrations. A really damp ski (e.g., a World Cup GS ski) will feel almost glued to the surface of the snow, even when skiing fast in firm, rough conditions. A ski that’s not damp (e.g., a lightweight touring ski) will get knocked around a lot, can feel “twitchy” in rough snow, and overall will require more skier input and focus to keep it tracking in the right direction while skiing fast in challenging conditions. Overall, heavier skis tend to be more damp than lighter skis. For more, see “suspension.””* |
| **14** | Deep snow | Definition not found. |
| **15** | Stable in crud | *“First you have untracked powder. Then you have fresh snow that gets chopped up, and after that chopped-up snow consolidates, gets warmed by the sun, and then hardens overnight, you have “crud.” Heavy, grabby, often hard & set-up clumps of snow. The heavier and wetter that “crud” gets, the more inclined we are to call it “mank.” If that set-up snow is colder & drier, that’s “crud.” Snow doesn’t necessarily need to warm and harden overnight to be considered crud; chop at the end of a resort powder day can often be considered crud once it’s been skied on by enough skiers, which is why we often include a section in ski reviews title “firm chop / crud.”* |
| **16** | Maneuverable | *“Maneuverable & Loose: We use these two terms fairly similarly, mostly to refer to how easily a ski lets you make a slarved, skidded, slashed, drifted turn. Very maneuverable or loose skis will not require much effort or focus to break them free from a carved turn, which can particularly be a big advantage in tight off-piste terrain (as well as deep snow), though this often comes at the expense of precision and edge hold on firm, smooth snow. We often use the term “loose” since it’s more specific to this ability to slide / skid / slarve / pivot, whereas “maneuverable” is a slightly more general term; we use the two mostly as synonyms, but “loose” leaves less room for ambiguity. More tip and tail taper, deeper rocker lines, reduced camber, increased degrees of reverse-camber, and softer flex patterns (torsionally & longitudinally) all tend to lead to a looser ski. A ski’s base and edge tune also play a significant role. In terms of how maneuverable a ski feels overall, you also have to factor in how quick / nimble / agile it feels (see below), among other things, which is why we always aim to describe how exactly a given ski feels “maneuverable.””* |
| **17** | Demanding | *“Demanding & Punishing: These two terms are closely related and generally refer to skis that aren’t all that easy to ski, particularly if you’re not a very experienced skier and/or not very physically strong. “Demanding” is a slightly broader term that can refer to both of those challenges, while we typically use the word “punishing” to describe skis that are very quick to discipline poor technique. A very heavy ski that’s also quite soft and rockered might be physically demanding to ski, but not punishing (e.g.,*[*J Skis Hotshot*](https://blisterreview.com/gear-reviews/2020-2021-j-skis-hotshot)*); a really stiff, minimally rockered, lightweight ski may not be very physically demanding to ski but could be very punishing (e.g.,*[*Atomic Vantage 97 Ti*](https://blisterreview.com/gear-reviews/ski-reviews/2018-2019-atomic-vantage-97-ti)*).”* |
| **18** | Tight terrain | *“This is a general term to describe off-piste terrain with lots of obstacles that require lots of shorter turns. We’ll often use “tight terrain” to refer to a ski’s performance in moguls, trees, steeps, and any other off-piste terrain that requires a similar skiing style with lots of on-the-fly adjustments and few long, sweeping turns.”* |
| **19** | Park-oriented | Definition not found. |
| **20** | Surfy/loose | See maneuverable above. |
| **21** | Stable on jumps | Definition not found. |
| **22** | Flotation | *“Generally, this refers to how well a ski rises in and stays above fresh, soft snow. A ski doesn’t need to stay all the way on top of powder to be fun in deep snow, but what’s particularly important is how maneuverable it is in these conditions. If a ski offers very poor flotation and sinks through deep snow, it will be difficult to turn. Or if its tips suddenly sink when you hit some deeper snow, it might pitch your forward and throw you “over the handlebars.” Another thing to keep in mind is that skis with more rearward mount points will generally offer more flotation from the front of the ski (with the longer front of the ski rising up and the shorter back of the ski sinking down); this allows for the skier to put more pressure on the front of the ski in deep snow. Skis with more centered mount points will generally require a more centered stance, particularly in deep snow, but can still “float” fairly well if you’re accustomed to or adapt to the differences in stance.”* |
| **23** | Deep chop | *“Powder after it’s been skied and cut up. Shallow chop (3-5” of tracked-out snow) isn’t much to contend with when the snow is light and fresh. But late in the day after a big storm, blasting through trenches and 12-24” deep pockets of powder can really test a ski’s stability — and your leg strength. That said, skis that are stable in chop (typically heavy and stiff ones) can make these conditions an absolute blast, since the snow is still fairly soft and forgiving, but you’ve also got limitless piles of chop to jump off. In reality, most “powder days” in a ski resort will consist of more chop skiing than untracked pow turns.”* |
| **24** | Refrozen chop | *“Crud / Mank: First you have untracked powder. Then you have fresh snow that gets chopped up, and after that chopped-up snow consolidates, gets warmed by the sun, and then hardens overnight, you have “crud.” Heavy, grabby, often hard & set-up clumps of snow. The heavier and wetter that “crud” gets, the more inclined we are to call it “mank.” If that set-up snow is colder & drier, that’s “crud.” Snow doesn’t necessarily need to warm and harden overnight to be considered crud; chop at the end of a resort powder day can often be considered crud once it’s been skied on by enough skiers, which is why we often include a section in ski reviews title “firm chop / crud.”*  *(And if it’s late in the season and you’re dealing with warm, slushy days and below-freezing nights, a similar freeze-thaw cycle can produce the dreaded “Coral Reef” or “Death Cookies,” which are basically the harshest, most extreme versions of crud.)”* |
| **25** | Lightest in the air | *“Swing weight : How heavy / sluggish or light / quick a given ski feels, particularly at its tips. A ski with a high swing weight will be more difficult to flick around from your ankles, which is most noticeable in the air or when skiing tight trees, steeps, and mogul runs. Skis with heavily tapered tips typically have lower swing weights because the widest (and heaviest) point on the ski is moved closer to the middle of the ski. Swing weight directly affects how nimble / agile / quick a ski feels.”* |
| **26** | Stable in chop | *“That said, skis that are stable in chop (typically heavy and stiff ones) can make these conditions an absolute blast […]”* |

# Key Terms Attributes Illustrated


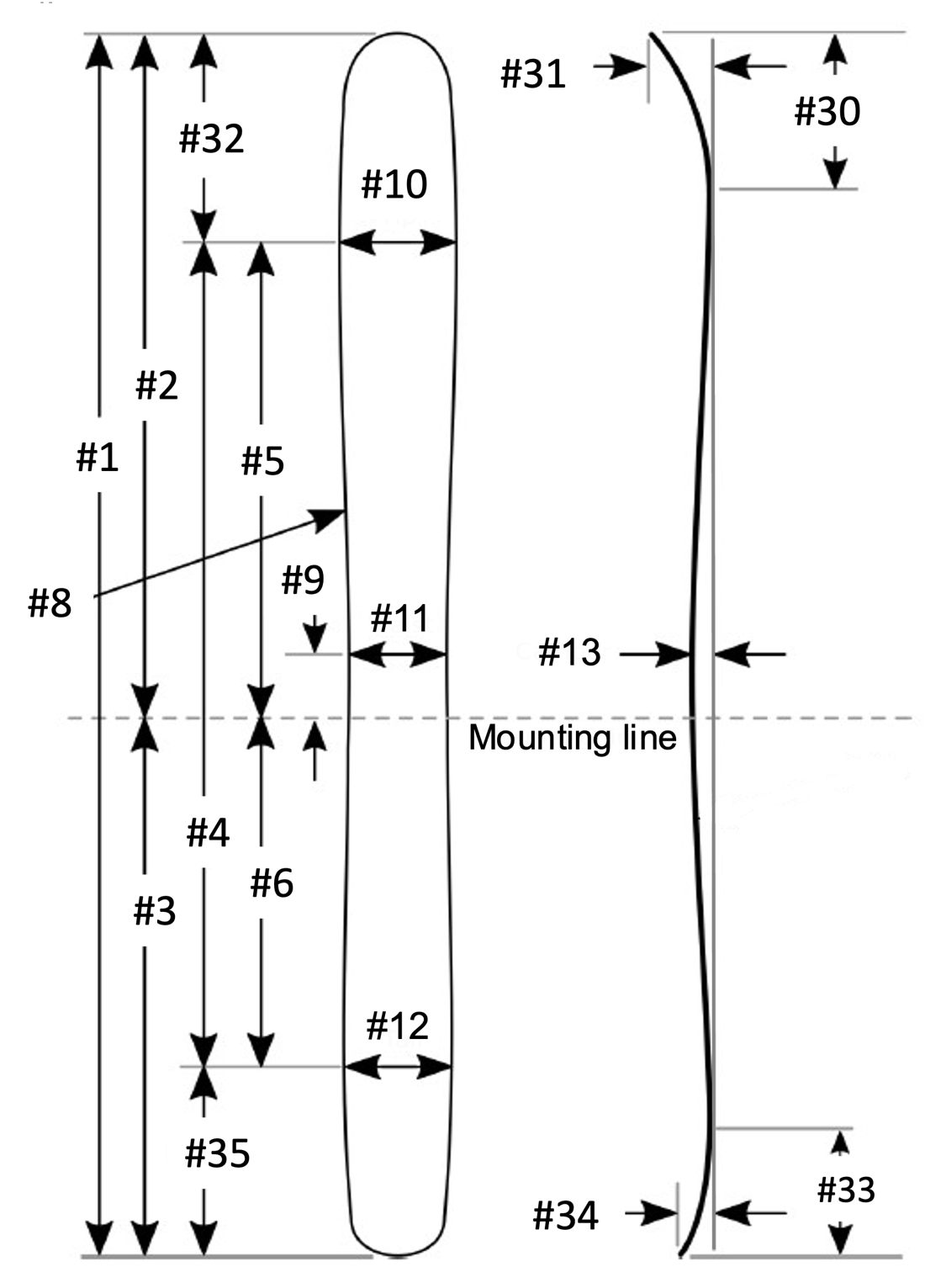


Figure 1: Ski geometrical attributes measured.
